# Supplementary figures and images for: Application of 23 Novel Serological Markers for Identifying Recent Exposure to Plasmodium vivax Parasites in an Endemic Population of Western Thailand
Source: Front Microbiol. 2021 Jun 29;12:643501. doi: 10.3389/fmicb.2021.643501 (PMC8279756; doi:10.3389/fmicb.2021.643501)

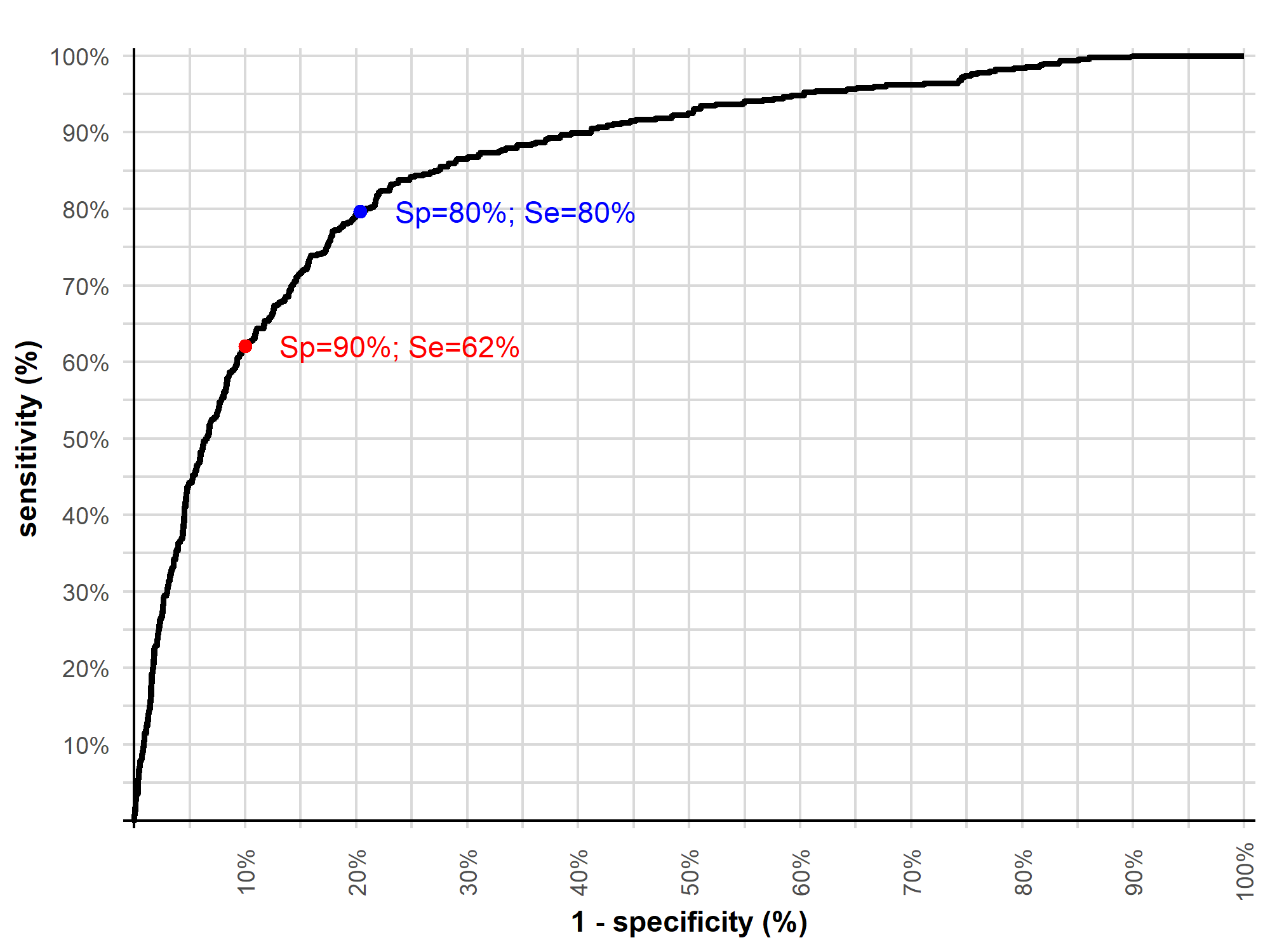

Supplement: Supplementary Figure 1 — Random Forest classification algorithm receiver operator characteristic curve (ROC). [file Image_1.PNG]

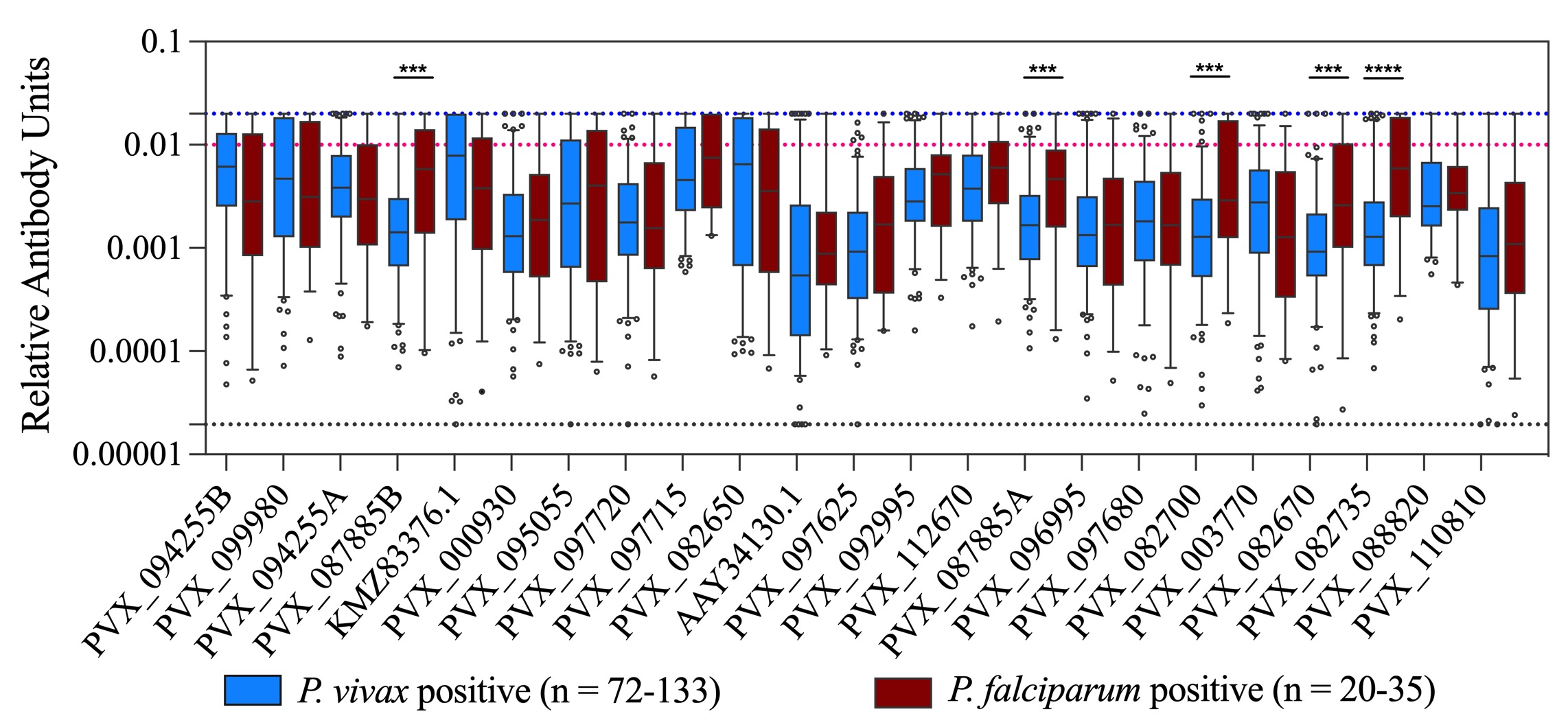

Supplement: Supplementary Figure 2 — Total IgG antibody levels against 23 P. vivax proteins in individuals with concurrent P. vivax monoinfections or concurrent P. falciparum monoinfections. Statistical difference was assessed using the Mann-Whitney rank test. ***p < 0.001, ****p < 0.0001. [file Image_2.JPEG]
